# Animal Poisoning: Toxins from Plants or Feed—An Important Chemical Risk for Domestic Animals

**DOI:** 10.3390/toxins16010039

**Published:** 2024-01-12

**Authors:** Andras-Laszlo Nagy

**Affiliations:** Department of Biomedical Sciences, Ross University School of Veterinary Medicine, Basseterre P.O. Box 334, Saint Kitts and Nevis; anagy@rossvet.edu.kn

Feed-, food-, water- and plant-related toxins are a major threat for animal and human health worldwide. These are natural toxins such as bacterial toxins, algal toxins, mycotoxins or phytotoxins and represent an important cause of disease in animals [1]. Phytotoxins and other feed-contaminating toxins are diverse and many of them have only recently emerged.

Companion animals are frequently exposed to chocolate, grapes, raisins, onions, decorative plants and cannabis products, while livestock are primarily exposed to phytotoxins and mycotoxins [2,3].

Many phytotoxins, especially the active compounds of decorative plants, have only emerged in recent years [4]. Climate change and the global trade in decorative plants are the main causes of this epidemiological trend [4,5,6].

In most countries in Europe, information regarding the incidence of poisonings, including those involving phytotoxins or feed-related toxins, are scarce to nonexistent due to a lack of efficient centralized reporting/poison control systems [4].

The aim of this Special Issue is to present new research regarding plant toxins and feed-associated toxicants, including the determination of phytotoxins and feed contaminants, the epidemiology of poisoning, innovative methods for the degradation of natural toxins, as well as descriptions of clinical signs and pathologic changes associated with exposure to different plant- and feed-related toxins.

We welcomed original research papers, short communications, case reports and review papers concerning the presented topics.

The topics covered by the published papers are diverse and include research regarding the occurrence of mycotoxins in cereal products (contribution 1); the determination of pesticide residues in animal feed (contribution 2); the biodegradation of gossypol (contribution 3); the identification of *Nerium oleander* using the PCR method (contribution 4); the toxin profile of different *Gymnodinium catenatum* strains from Iberian coastal waters (contribution 5); and hypoglycin A poisoning in herbivores (contribution 6).

Macri et al. (contribution 1) reported results from monitoring type A and B trichothecenes in cereal products sold in Romanian markets. The authors used gas chromatography–mass spectrometry (GC-MS) analysis and identified thirteen type A and type B trichothecenes in wheat, bread and bakery products and pasta commercialized in Romania. According to this research, from the total of 121 samples analyzed, 90.08% were contaminated with trichothecenes, and deoxynivalenol was the predominant mycotoxin in all categories of products. In this study, significant statistical differences were observed regarding the presence of various mycotoxins in different cereal products. The authors observed significant differences between samples coming from different geographical regions of the country.

Xu and Murphy (contribution 2) presented a new simple and fast method developed for the simultaneous determination of pyrethrins, pyrethroids and piperonyl butoxide in livestock and poultry feeds. In this research, the authors used a liquid chromatography–tandem mass spectrometry method for quantitative determination of the contaminants. The researchers concluded that this new method can be a valuable tool in animal health and food safety diagnostic applications. It would also be useful for veterinary toxicology investigations concerning pyrethrin-related feed contamination.

Another interesting paper (contribution 3) presented the biodegradation of free gossypol by recombinant *Helicoverpa armigera* carboxylesterase expressed by the yeast *Pichia pastoris*. Using this method, the authors managed to effectively degrade free gossypol with a degradation rate of 89%. According to the authors, the study confirms that recombinant carboxylesterase isolated from *H. armigera* can be utilized as an effective gossypol-degrading enzyme for cottonseed meal, thus allowing these products to be used as animal feed.

In their paper, Bai et al. (contribution 4) described a rapid and accurate identification method for *Nerium oleander* using a quantitative real-time PCR (qPCR) method. The authors designed and validated a new pair of oleander-specific primers, JZT-BF/BR, which could identify oleander in forage and food mixtures. In this study the qPCR was capable of accurate authentication even at a low DNA concentration of 0.001 ng/μL. The results of the research offer a new, efficient tool for the detection of oleander in accidental and intentional poisoning cases in humans and animals. The authors emphasized the importance and potential future application of the qPCR method in toxin identification and the diagnosis of poisonings.

A study performed in Portugal (contribution 5) described the toxin profile of two strains of *Gymnodinium catenatum*, the main species responsible for paralytic shellfish poisoning events along the Portuguese coast. In this study, the evaluation of the toxin profile was realized using a high-performance liquid chromatography with fluorescence detection (HPLC-FLD) method. The study revealed that the two studied strains contained a higher percentage of decarbamoylsaxitoxin (dcSTX) and a lower amount of N-sulfocarbamoyl gonyautoxin 1 and 4 (C3,4) compared to the other strain from the region, and low levels of the toxins decarbamoyl neosaxitoxin (dcNEO), gonyautoxin 1 and 4 (GTX1,4) and neosaxitoxin (NEO). The authors concluded that the study is relevant for the characterization of the toxin profile of *G. catenatum* and emphasized the potential characteristic biogeographic profile of different strains directly affecting their toxicity. 

In another research paper, Renaud et al. (contribution 6) surveyed hypoglicin A (HGA) exposure from *Acer pseudoplatanus* in different herbivorous species that, until now, had not been described as being at risk. The authors hypothesized that any herbivore pasturing around this tree may be at risk for HGA poisoning. The major focus of the study was animals in zoological parks. The authors showed that proximal fermenter species are less susceptible to HGA poisoning due to ruminal transformation of the toxin. They suggest that, in ruminants, a gradual alteration of fatty acid metabolism in the case of HGA poisoning can lead to subclinical cases and reported an increase in serum acylcarnitines in animals with detectable HGA or methylenecyclopropylacetic-carnitine in their blood, which indicates a gradual alteration of fatty acid metabolism. The authors concluded that *Acer pseudoplatanus* is responsible for clinical poisoning in camels, Père David’s deer and gnus, and the pathophysiology of the disease is similar to that described in equine atypical myopathy. 

This Special Issue includes three review papers that discuss emerging and non-emerging plant intoxications in domestic animals in Europe (contribution 7); the toxicity of house plants to companion animals (contribution 8); and co-exposure to mycotoxins and their importance for the appearance of foodborne ailments and diseases in humans and animals (contribution 9).

In their study, Nagy et al. (contribution 7) presented an update regarding emerging plant poisonings in domestic animals in Europe. The authors described the main plant species involved in domestic animal poisoning in Europe, the main toxins from these plants, their mechanism of action and the main clinical signs induced by the toxicosis. The authors emphasized that future research should focus on the characterization of the toxicity of emerging phytotoxins and highlighted the necessity of a centralized reporting system for animal poisoning at the European level.

In her review article, Siroka (contribution 8) described the most common indoor toxic plants grown in Europe, including the mechanisms of action of their active substances and the clinical signs associated with exposure. The manuscript contains numerous pictures of the described plants. General therapeutic protocols used in plant poisonings are also included in the paper. The author emphasizes the importance of toxic plant identification, and in line with this recommends continuous education of veterinarians and the public. The review highlights the importance of preventive measures like correct and mandatory labeling of plant species and description of their potential toxic effects.

In his manuscript, Stoev (contribution 9) evaluated the hazard posed by mycotoxin co-contamination of animal feeds and human foods in the appearance of foodborne ailments and diseases. Hygiene control and risk assessment measures are also discussed in the manuscript. The author highlighted the importance and necessity of harmonizing mycotoxin regulations and food safety control measures to facilitate interstate food trade and to ensure global food safety.
